# Modulation of the nanoscale motion rate of *Candida albicans* by X-rays

**DOI:** 10.3389/fmicb.2023.1133027

**Published:** 2023-03-21

**Authors:** Maria N. Starodubtseva, Irina A. Chelnokova, Nastassia M. Shkliarava, María Inés Villalba, Dmitry V. Tapalski, Sandor Kasas, Ronnie G. Willaert

**Affiliations:** ^1^Department of Medical and Biological Physics, Gomel State Medical University, Gomel, Belarus; ^2^Laboratory of Bionanoscopy, Institute of Radiobiology of NAS of Belarus, Gomel, Belarus; ^3^Laboratory of Biological Electron Microscopy, École Polytechnique Fédérale de Lausanne (EPFL), University of Lausanne (UNIL), Lausanne, Switzerland; ^4^Department of Microbiology, Gomel State Medical University, Gomel, Belarus; ^5^Centre Universitaire Romand de Médecine Légale, Unité Facultaire d’Anatomie et de Morphologie (UFAM), University of Lausanne (UNIL), Lausanne, Switzerland; ^6^International Joint Research Group VUB-EPFL NanoBiotechnology & NanoMedicine (NANO), Vrije Universiteit Brussel and École Polytechnique Fédérale de Lausanne (EPFL), Lausanne, Switzerland; ^7^Alliance Research Group VUB-UGent NanoMicrobiology (NAMI), Research Group Structural Biology Brussels, Vrije Universiteit Brussel, Brussels, Belgium

**Keywords:** optical nanomotion detection, *Candida albicans*, X-rays, antifungal drugs, fluconazole, spectral analysis

## Abstract

**Introduction:**

Patients undergoing cancer treatment by radiation therapy commonly develop *Candida albicans* infections (candidiasis). Such infections are generally treated by antifungals that unfortunately also induce numerous secondary effects in the patient. Additional to the effect on the immune system, ionizing radiation influences the vital activity of *C. albicans* cells themselves; however, the reaction of *C. albicans* to ionizing radiation acting simultaneously with antifungals is much less well documented. In this study, we explored the effects of ionizing radiation and an antifungal drug and their combined effect on *C. albicans*.

**Methods:**

The study essentially relied on a novel technique, referred to as optical nanomotion detection (ONMD) that monitors the viability and metabolic activity of the yeast cells in a label and attachment-free manner.

**Results and discussion:**

Our findings demonstrate that after exposure to X-ray radiation alone or in combination with fluconazole, low-frequency nanoscale oscillations of whole cells are suppressed and the nanomotion rate depends on the phase of the cell cycle, absorbed dose, fluconazole concentration, and post-irradiation period. In a further development, the ONMD method can help in rapidly determining the sensitivity of *C. albicans* to antifungals and the individual concentration of antifungals in cancer patients undergoing radiation therapy.

## Introduction

The optical nanomotion detection (ONMD) technique was recently developed to study the nanomotion pattern of single cells as well as cellular populations (Willaert et al., 2020). The technique is simpler than its atomic force microscopy (AFM)–based counterpart (Longo et al., 2013) since it is easily accessible and most importantly label- and attachment-free. It was essentially developed to perform rapid antifungal susceptibility tests on yeast (Willaert et al., 2020; Radonicic et al., 2022). Recent experiments using the AFM- and optical-based nanomotion detection method demonstrated that cellular nanomotion is directly linked to the metabolic activity of the cell. Cell metabolic activity changes of several strains of *Candida* (*C. albicans DSY294*, *C. glabrata DSY562*, and *C. lusitaniae DSY4606*) in ethanol-induced life-death transition have been studied by observing cellular nanomotions (Willaert et al., 2020). Concentration- and time-dependent effects of several antifungals (amphotericin B, caspofungin, and fluconazole) on the *C. albicans* nanomotion was investigated using both the AFM- and optical-based nanomotion detection techniques (Kasas et al., 2015; Willaert et al., 2020).

It is known that ionizing radiation influences cellular metabolic activity. The changes in metabolic activity after yeast X-ray irradiation were observed for *C. guilliermondii* (35 kV, 300 Gy) (Navasardyan et al., 2017). The exposure of cells and tissues to ionizing radiation leads to oxidizing events that change the structure of biologically important molecules. The changes are induced by the direct interaction of ionizing radiation with the target macromolecules or *via* the products released by the radiolysis of water. These early events induce, among others, oxidative stress that leads to cellular metabolic and proliferative changes that ultimately can result in cellular death (Azzam et al., 2012). Cancer is nowadays a major public health problem at the planetary scale. Among the therapeutic options to fight this disease, radiotherapy is widely used alone or in combination with other treatments to cure or reduce the risk of cancer recurrence. One of the acute side effects of radiation therapy is candidiasis. As an illustration, head and neck cancer patients undergoing radiation therapy often develop oral candidiasis over the course of their treatment (Mañas et al., 2012; da Silva et al., 2017). After the radiation therapy starts Candida colonization can be observed in about 75% of patients. Local manifestations of fungal infection occurred in 26% of colonized patients (Pinel et al., 2012). Nowadays the mechanisms underlying radiotherapy-induced candidiasis are not clear. Radiation-induced free radicals and DNA damage modify various cellular signaling pathways regulating cell proliferation, differentiation, and death that cause inflammation and activate apoptosis (Ingrosso et al., 2021).

*Candida albicans* is an opportunistic pathogenic microorganism that co-exists in humans as a commensal without harming the host, but if the immune status of the host organism or its microbiota is violated, which is the case with radiotherapy, it can cause extensive colonization of the mucous membrane and local or systemic disease (Casadevall and Pirofski, 2007; Costa-de-Oliveira and Rodrigues, 2020). Gordee and Simpson (1967) showed that the pre-irradiation by X-rays significantly reduced the number of *C. albicans* cells required for the LD50 of experimentally infected mice (Gordee and Simpson, 1967). On the other hand, ionizing radiation influences *Candida* cells themselves, which can potentially modify their virulence and sensitivity to antifungal drugs. *In vitro* studies demonstrate that ionizing radiation can either inhibit *Candida* proliferation or it can potentiate its virulence, the outcome depending on the source of irradiation, *Candida* strain, and culture conditions (Ueta et al., 2001; Ben-Yosefa et al., 2005; Cagnacci et al., 2008). It should be emphasized that ionizing radiation effects strongly depend on the phase of the cell cycle. Budding yeast cells in S and M phases are known to be more resistant to radiation than non-budding cells in the G1 phase (Beam et al., 1954; Langguth and Beam, 1973). But proliferating yeast cells predominantly in S/G2 phase exhibit greater X-ray-induced chromosomal loss and DNA deletion than stationary (G1/G0)-phase yeast cells (Parry et al., 1979; Hafer et al., 2010).

The effect of antifungal drugs on the cellular nanomotion of yeast have been explored in several recent studies; however, how ionizing radiation influences yeast nanomotion as well as the combined effect of radiation and antifungal drugs on yeast nanomotion has not yet been studied. Therefore, we investigated the effect of X-ray radiation and an antifungal separately and in combination on the nanomotion of *C. albicans* cells using the ONMD method. The results obtained can help in further understanding the mechanisms of candidiasis pathogenesis and the development of strategies to determine the sensitivity of *C. albicans* to antifungal drugs during radiotherapy.

## Materials and methods

### Strains and cell growth

*Candida albicans* (ATCC 10231) were grown on Sabouraud glucose-peptone agar, HiMedia Laboratories, India (40 g/L glucose, 10 g/L peptone, 15 g/L agar) for 2 days at a temperature of 28°C. Several colonies of the same type from Sabouraud agar were transferred with a cotton swab into a test tube with 10 mL of Sabouraud broth. Under the control of a densitometer, the turbidity of the suspension was adjusted to 10 McFarland, and the estimated concentration of the cell suspension was 10^8^ cells/ml. This concentration of cells was experimentally selected to be optimal for microscopy when 100–150 *Candida* cells were observed in one field of view.

*Candida albicans* ATCC 10231 is a fluconazole (FLC) susceptible strain (Silva et al., 2022). A stock solution of FLC (10 mg/mL) (Pharmland) was prepared in sterile distilled water. The FLC solution was introduced into vials containing *C. albicans* suspensions in Sabouraud broth to achieve a concentration of 10 or 1000 mg/L. The FLC concentration of 10 mg/L corresponds to 20 × MIC (minimum inhibitory concentration) and 0.625 MFC (minimum fungicidal concentration), and the concentration of 1000 mg/L corresponds to 2,000 × MIC and 62.5 MFC (Silva et al., 2022).

After irradiation, the vials were incubated on an orbital shaker incubator (ES-20, Biosan, Latvia) at 28°C and 180 rpm. This was supposed to slow down the adhesion of cells to the plastic of the vial. Before performing the ONMD measurements (points 3–24 h), the suspension was transferred to a sterile tube and diluted with Sabouraud broth until a concentration of 10^8^ cells/ml (10 McFarland).

### X-ray irradiation

The *C. albicans* suspension was placed in ventilated vials for cell cultures (cell culture flask T-25, standard surface, with filter cap, Sarstedt, Germany). The suspension of cells in vials had the form of a flat layer 2–3 mm thick at room temperature. The vials were exposed to X-ray irradiation using the X-RAD 320 biological irradiation unit with a tube voltage of 320 kV, a current of 12.5 mA with a power of 5 Gy/min, SSD of 40 sm, filter 1 (2 mm Al). The controlled experimental conditions were as follows: the temperature was 20.9 ± 0.7°C and the humidity was 62.5 ± 2.5%. As a reference, non-irradiated samples (untreated control) were used, which were kept under the same experimental conditions as the irradiated samples.

The irradiation of the cell suspensions was carried out in accordance with two experimental protocols. In protocol 1, the *C. albicans* suspensions (5 ml) were irradiated in fractions of 40 min with a dose of 200 Gy accumulated during each fraction, with a break between fractions of 5 min. A total of 4 fractions of irradiation were carried out, the absorbed dose after each fraction of irradiation was 200, 400, 600, and 800 Gy (the cell morphology of the control and the irradiated sample is shown in Supplementary Figure 1). The first protocol was used in the study of the effect of X-rays on yeast colony formation and yeast growth kinetics. In protocol 2, the *C. albicans* suspensions (7 ml) with or without the addition of FLC at concentrations of 10 and 1000 mg/L were irradiated by X-rays in the absorbed doses of 300 and 600 Gy. At the dose rate of 5 Gy/min, the process of the irradiation of the cell sample to reach the absorbed dose of 600 Gy took 2 h. To exclude the differences in incubation time before movie recording, the cell samples with the absorbed dose of 300 Gy, control cell samples and cell samples with only FLC treatment were kept under controlled temperature and humidity conditions for 2 h. The period after the start of the experiment and before the first record of ONMD movies was 2 h 20 min for all samples. The second protocol was used in the experiments using the ONMD method.

### Yeast colony formation and yeast growth

The plated cells were incubated for 48 h at 28°C, after which the number of colonies with a diameter of 1.0–1.5 2-3 mm was counted. Further, pure cultures from small colonies were accumulated, and their identification was carried out to exclude contamination. The resulting strains were placed in Sabouraud broth with the addition of 30% (v/v) glycerin and frozen at −62°C for long-term storage.

To assess the growth kinetics of pure cultures grown on Sabouraud agar for 2 days at 28°C, suspensions were prepared in a sterile isotonic solution of sodium chloride with an optical density (the absorbance) of 10 McFarland. The resulting suspensions were introduced into glass tubes with Sabouraud broth until an optical density of 0.5 McFarland (densitometer control) was reached, and the estimated concentration was 5 × 10^6^ cells/ml. The cells were incubated in an ES-20 incubator shaker (Biosan, Latvia) at 35°C with orbital stirring of 200 rpm. The optical density was measured at 600 nm every 30 min to determine the biomass of the yeast. The non-irradiated culture served as a control. Afterward, the early stage of growth kinetics (the first 4 h) was fitted by the exponential function:


(1) $$O\,D_{600}=a+b\,e^{R_{0}\,t}$$

where *OD*_600_ is the optical density at 600 nm depending on the total amount of biomass at time *t*, *a* is the initial *OD*_600_, parameter *R*_0_ is related to the yeast growth rate, and *b* is an arbitrary constant. The parameters *a*, *b*, and *R*_0_ were determined by fitting the experimentally obtained data (OriginPro software).

The dependence of parameter *R*_0_ on the absorbed dose (D) was fitted by the exponential function:


(2) $$R_{0}=c+k\,e^{-\frac{D}{d}}\,\ldots \,\ldots \,\ldots \,\ldots \,\ldots \,\ldots \,\ldots \,\ldots$$

where *c, k*, and *d* are arbitrary constants determined by fitting the experimentally data (OriginPro software).

### Optical nanomotion experiment

**FIGURE 1:**
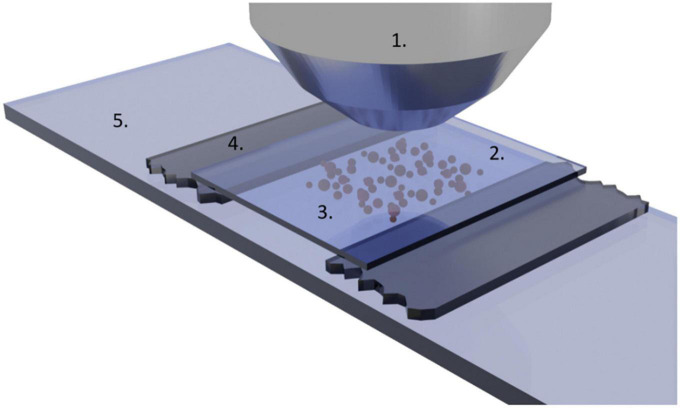
Experimental setup for optical nanomotion detection. (1) Microscope objective, (2) coverslip, (3) analysis chamber with yeast cells (not at scale), (4) 150-micrometer high double face rubber tape, and (5) sample holder glass.

Twenty μl of the yeast cell suspension was dispensed onto a glass slide (MiniMed, Russia) between two Scotch tapes fixed parallel to each other and perpendicular to the longer side of the slide, then covered by a coverslip (Figure 1). Scotch tapes in such construction create an imaging chamber with a thickness of 150 μm. To stabilize the cell movement state in the chamber, the yeast cells were allowed to sediment for a period of 10 min before starting the measurement. The motion of cells was observed by taking movies (Supplementary Video 1) of 300 frames with a framerate of about 6 frames per second (fps) using a MiniMed 502 microscope with a 40× objective, a BestScope camera, and the TC-Capture software. Recordings were done at room temperature and humidity of 45 ± 5 %.

### Nanomotion data processing

The ONMD algorithm described previously was used for data processing (Willaert et al., 2020). The main part of the program is based on the algorithm of Guizar-Sicairos et al. (2008) work. The software calculates the cell displacement for each frame. The data of the cell displacements were represented as the following data set:


(3) $${\left(X,Y\right)}_{i,j}=\left(\begin{array}{cc} x_{11} & y_{11}\\ \ldots & \ldots \\ \begin{array}{c} \begin{array}{c} x_{1\,n} \end{array}\\ \begin{array}{c} x_{21} \end{array} \end{array} & \begin{array}{c} \begin{array}{c} y_{1\,n} \end{array}\\ \begin{array}{c} y_{21} \end{array} \end{array}\\ \ldots & \ldots \\ x_{k\,n} & y_{k\,n} \end{array}\right)$$

where *i* was an adjacent number of cell in a sample (*i* = 1, *k*; k varied from 5 to 30), *j* was an adjacent number of a frame for each cell in a sample (*j* = 1, n). For each cell the average rate of cellular nanomotion for a certain Δ*t* was calculated using the Euclidean distance *S*_*i,j*_ between two cell positions adjacent in time and time interval between two adjacent frames Δ*t*:


(4) $$v_{i,j}=\frac{S_{i,j}}{\Delta \,t}=\frac{\sqrt{{\left(x_{i,j}-x_{i,j+1}\right)}^{2}+{\left(y_{i,j}-y_{i,j+1}\right)}^{2}}}{\Delta \,t}.$$

The average time interval was Δ t = 0.21 ± 0.02 s. Based on the obtained results data sets (*t,V*)_*i,j*_ were formed for each cell sample. The nanomotion cell rate was measured in nm/10 ms. A discrete Fourier transform was performed to the data set (*t,V*)_*i,j*_ using Fast Fourier Transform (FFT) option of OriginLab software. Discrete Fourier transform is the basis for many signal processing procedures. It converts a signal from the time domain into the frequency domain. For further analysis, we used RMS amplitude frequency spectrum. Image J software was used to determine the cell diameter using light microscopy images. The diameter of G0-phase cells normally having a spheroidal shape was determined as the average of their maximal and minimal sizes. The diameter of budding cells (in S/G2 phase of the cell cycle) having the shape of two linked spheroids were roughly approximated as a diameter of one spheroidal cell times 1.3.

### Statistical analysis

Statistical analysis of experimental data was performed using OriginPro, version 2019b and Statistical calculator ‘‘Statistical Kingdom.’’^1^ The data were checked for compliance with the normal distribution law using the Kolmogorov-Smirnov test. The data are represented as either the median and limits of the interquartile range [Me(LQ; UQ)] or the mean and the limits of 95% confidence interval (95% CI). Multiple comparison analysis was performed using Kruskal-Wallis ANOVA by ranks test with Bonferroni’s correction and Dunn’s test.

## Results

### The effect of X-rays on the *C. albicans* colony formation and growth

**FIGURE 2:**
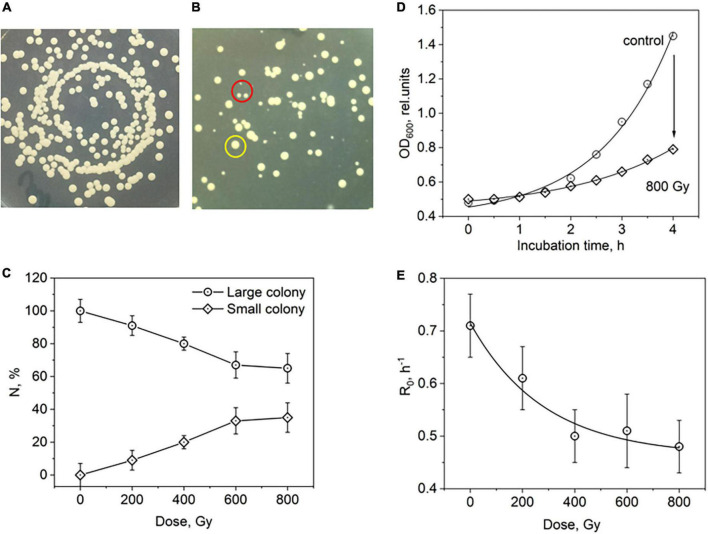
X-ray absorbed dose effect on *Candida albicans* colony formation and growth parameters. **(A,B)** X-ray induced changes in the *C. albicans* colony formation. **(A)** Control sample (non-irradiated control sample), **(B)** yeast cell sample after irradiation by X-rays a dose of 800 Gy. Two types of yeast colonies, large (yellow circle) and small (red circle), are presented in the sample. **(C)** The percentage of two types of yeast colonies depending on the absorbed dose. **(D)** The dependence of *C. albicans* biomass on the adsorbed dose of X-ray radiation for the non-irradiated control sample and cells exposed to X-rays (800 Gy). The yeast biomass was assessed using the absorbance (OD) at 600 nm. The kinetic curves of yeast growth were fitted with exponential function: *OD*_600_ = *a* + *be*^*R*_0_*t*^, *R*^2^>0.994. Parameter R_0_ is related to yeast growth rate and measured in h^– 1^. **(E)** The X-ray induced decrease in the yeast growth rate. The dose-dependence of yeast growth rate was fitted with exponential function: *R*_0_ = 0.46273 + 0.252*e*^−(*x*/282.57)^, *R*^2^ = 0.961, *d* = 282.57 Gy.

In the first step, we studied the effect of X-rays on *C. albicans* colony morphology and growth rate. It permitted us to determine the X-ray doses to use in further ONMD experiments. The colonies that were not exposed to X-rays displayed a typical morphology, i.e., a diameter of 2–3 mm with a hemispherical shape and smooth edges. The samples irradiated by X-rays displayed two different types of colonies: colonies with typical morphology and small colonies with a diameter of about 1 mm (Figures 2A, B). By increasing the absorbed dose in the range of 0–800 Gy, a decrease in the number of colonies with typical morphology and an increase in the number of small colonies was observed (Figure 2C).

Ionizing radiation is known to inhibit cell growth and cell division. We studied the yeast growth kinetics at the early stage of growth during the first 4 h (Figure 2D). The growth kinetics at this stage is well fitted by an exponential curve. The coefficient *R*_*o*_ in the exponential function reflects the growth rate of the yeast biomass. With an increase in the absorbed dose in the dose range up to 800 Gy, this coefficient decreases exponentially (Figure 2E). Both obtained dose-dependent kinetics for the number of small colony appearance and the growth rate decrease revealed the quick change of the parameters in the range of 0–400 Gy and their relative stabilization in range of 600-800 Gy (Figures 2C, E). For the next experiments, we used an absorbed dose of 600 Gy as relative a high dose (HD) and a dose of 300 Gy as a relative low dose (LD).

### The effect of X-rays and antifungal drug on the nanomotion of *C. albicans*

**FIGURE 3:**
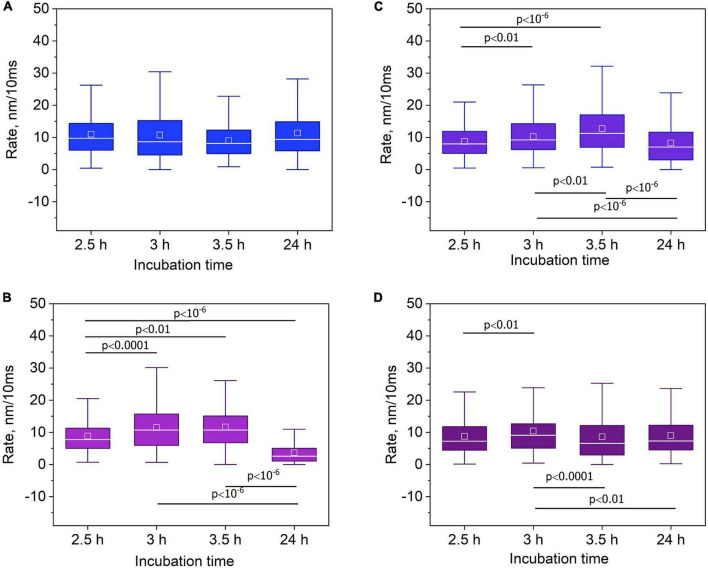
The effect of X-ray irradiation and antifungal drug treatment on the nanomotion rate of *Candida albicans*. The absorbed dose was 600 Gy, the concentration of FLC was 1000 mg/L. **(A)** The absence of significant changes in the nanomotion rate for the control cell sample (non-irradiated and untreated) (Multiple comparison (Dunn’s test) *post-hoc* analysis with Bonferroni’s correction: *p* > 0.008333). **(B)** Time-dependent behavior of the nanomotion rate for the irradiated cell samples. ANOVA by ranks test (*p* < 10^– 6^); multiple comparison (Dunn’s test) *post-hoc* analysis. **(C)** Time-dependent behavior of the nanomotion rate for the FLC-treated cell samples. ANOVA by ranks test (*p* < 10^– 6^); multiple comparison (Dunn’s test) *post-hoc* analysis. **(D)** Time-dependent behavior of the nanomotion rate for the FLC-treated and irradiated cell samples. ANOVA by ranks test (*p* = 0.006913); multiple comparison (Dunn’s test) *post-hoc* analysis. Data are represented as median (line), mean (square), IQR (box), and 1.5 IQR (whiskers).

In a next step, we studied *C. albicans* nanomotion after irradiation with X-rays, FLC treatment and the combination of the two factors. To assess the effect of the incubation time after irradiation or FLC treatment we monitored the cellular oscillations at 2.5, 3, 3.5, and 24 h after the exposure. The nanomotion of control yeast cells was revealed not to be time dependent, at least within a 24 h period (Figure 3A). In the case of irradiation by HD (600 Gy) X-rays, the nanomotion depended on the incubation time, with a maximal value at 3–4 h (Figure 3B). By the end of the 24-h incubation period, the nanomotion activity of yeast cells decreased to 5.3 (3.6, 8.0) times compared to the maximal observed value at 3.5 h and to 3.7 (2.5, 6.4) times as compared to control values (Kruskal-Wallis ANOVA by ranks test, Dunn’s test *post-hoc* analysis: *p* < 10^–6^).

A similar nanomotion pattern was observed after treatment with FLC at high concentration (1000 mg/L) (Figure 3C). The maximal nanomotion activity was reached after 3.5 h following the antifungal treatment. By the end of a 24-h period the nanomotion significantly decreased to 1.6 (1.5;2.3) times compared to the observed value at 3.5 h (Kruskal-Wallis ANOVA by ranks test, Dunn’s test *post-hoc* analysis: *p* < 10^–6^). After the combined action of the two factors, i.e., radiation and antifungal treatment, the kinetic curve did not present a clear maximum and a decrease in the nanomotion rate started after 3 h (Figure 3D); importantly, in a lower extent as compared to either irradiation or FLC treatment alone.

Our results and literature data show that the nanomotion rate is time-dependent and dose-dependent when the cells are exposed to X-rays, and time-dependent and concentration-dependent when the cells treated with antifungal drugs (Willaert et al., 2020). Using a relatively low (10 mg/L) and relatively high (1000 mg/L) concentration of FLC, we studied the *C. albicans* nanomotion after exposure to X-rays at the absorbed dose of 300 and 600 Gy at a fixed incubation time (3.5 h) (Supplementary Figure 2 and Supplementary Table 1). A low radiation dose influenced insignificantly the nanomotion in case of the absence of FLC. At low FLC (10 mg/L) concentration, the X rays slightly inhibited the nanomotion of yeast cells. At high FLC concentrations and a dose of 300 Gy the distribution of the nanomotion rates showed two peaks: one peak was close to the control values (about 8–9 nm/10 ms) and another one was shifted to the range of lower values (about 6–7 nm/10 ms). A high radiation dose intensified the nanomotion as compared to a lower dose in control cell samples. FLC supports to inhibition of the HD radiation-induced elevated level of nanomotion.

To better characterize the obtained nanomotion signals we carried out spectral analysis, which reflects the structure and the nature of the oscillating cells (Jelínek et al., 2009; Wohl and Sherman, 2021). The analysis permitted identifying frequencies that significantly decreased after 24 h upon X-rays irradiation. Spectral analysis was performed for *Candida* cells in two different phases of the cell cycle. Non-budding yeasts were classified as G1 or G0 phase and budding yeasts as S or G2 phase. A high percentage of cells were in the S/G2 phase of the cell cycle (70%) 24 h after the start of the experiment. Initial cultures at time zero were comprised predominantly (>90%) of G1/G0-phase cells, which agrees with literature data on *S. cerevisiae* (Hafer et al., 2010). From the nanomechanical point of view, the oscillatory behavior of non-budding and budding yeast cells is expected to be different because of the difference in the mass of single cells in the G1/G0 phase (one cell) and in S/G2 phase (two new cells). It should be pointed out that all results presented in the previous paragraph were obtained using yeast cells in G1/G0 phase.

**FIGURE 4:**
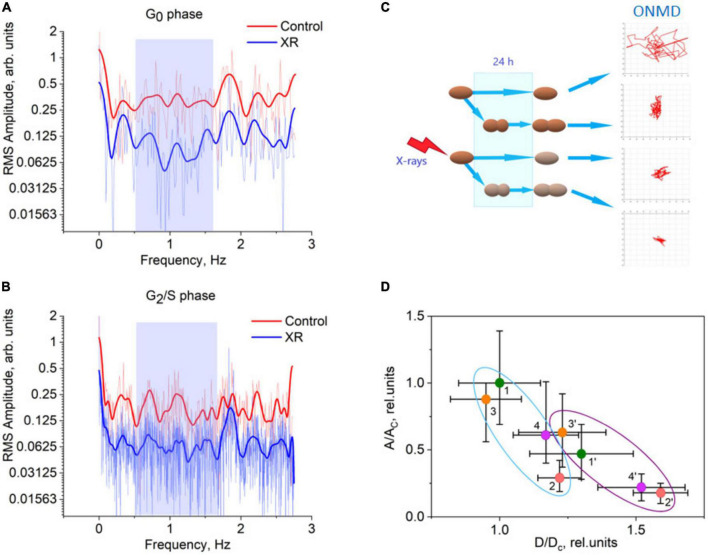
Spectral analysis of *Candida albicans* nanomotion rate at 24 h incubation time after experiment start. **(A,B)** Fourier transform spectrum for the RMS amplitude of the nanomotion rate. G0-phase yeast cells **(A)** S/G2-phase yeast cells **(B)**. The blue rectangle in the figures shows the frequency range of 0.55–1.60 Hz that lies between two Fourier spectrum peaks that is characteristic for all the *C. albicans* samples studied (about 0.3 and 1.9 Hz). Pale curves represent experimental data, bright colored curves represent the results of the curve smoothing by the FFT filter method. **(C)** The scheme of the analysis of cell nanomotion data for control (non-irradiated and untreated) and X-ray irradiated cultures. **(D)** The relative RMS amplitude of cell nanomotion rate averaged over the frequency range of 0.55–1.60 Hz against the relative averaged cell diameter for *C. albicans* after their exposure to various factors. Relative RMS amplitude was calculated with respect to the median RMS amplitude of the control yeast culture. *A* is the RMS amplitude of the studied sample, *A*_*c*_ is the RMS amplitude of the control (non-irradiated and untreated) sample. Relative cell diameter was assessed using light microscopy images and ImageJ software and calculated with respect to the median cell diameter of the control yeast culture. *D* is the cell diameter in the studied *C. albicans* sample, *D*_*c*_ is the cell diameter of the control *C. albicans* culture. The diameter of cells normally having spheroidal shape was assessed as the average of their maximal and minimal sizes. The diameter of budding cells having the shape of two linked together spheroids is roughly approximated as the diameter of spheroidal cell times 1.3. The cell samples were marked using the following numbers and colors: control (1, 1′, green circles), X-ray irradiated (2, 2′, scarlet circles), FLC-treated (3, 3′, orange circles) and FLC-treated and X-ray irradiated (4, 4′, lilac circles), where 1, 2, 3, and 4 (included into light blue oval) represent the data for G0-phase cells, 1′, 2′, 3′, and 4′ (included into violet oval) represent the data for G2/S-phase cells.

Figure 4 displays the nanomotion spectral parameters after 24 h exposure as a function of the cell cycle. The scheme of the analysis of cell nanomotion data for the control and X-ray irradiated cultures is represented in Figure 4C. For cells in the G0 phase (Figure 4A), a deep dip at frequencies of 0.55–1.60 Hz, which appeared in the spectrum of cell oscillation RMS amplitudes after irradiation with X-rays, attracts attention. As one would expect for budding cells, the oscillation amplitudes of the control and the irradiated culture were lower for almost all analyzed frequencies (Figures 4A, B). This frequency range coincides with the one that was mentioned previously as a “cell activity region” for cell nanomotion (Willaert et al., 2020). Because the different factors differently influenced the cell morphology, we analyzed the relationships between RMS amplitude averaged over the frequency range of 0.55–1.60 Hz and the averaged cell diameter (Figure 4D). In general, budding cells (points 1′-4′) oscillate with lower amplitude at the nanoscale compared to no-budding cells (points 1–4). But for FLC-treated cells, the difference between amplitudes for two cell cycle phases is absent (points 3-3′). A similar situation is observed for X-ray irradiated cells (points 2-2′). For the combined action of X-rays and the antifungal drug, the difference between the amplitudes of budding and non-budding cells was significant (points 4′-4) and like the ones observed for the control culture (points 1′-1).

## Discussion

In the first part of this work, we studied the time-dependent changes of nanomotion of *C. albicans* cells after exposure to X-rays. In classical radiobiology, the effect of ionizing radiation on cells is characterized by their survival and proliferation potential. The dependence of *C. guilliermondii* yeast cell survival fraction after X-ray exposure on the absorbed dose has been shown to be represented as a sigmoid curve (Navasardyan et al., 2017). The sigmoid survival curve indicates the presence of a well-developed mechanism of radiation-induced damage repair in cells. With an increase in the radiation dose, the effectiveness of the repair system decreases due to damage to the repair system itself. The *Candida* yeast species are more radio-resistant compared to other yeast cells such as for example *Saccharomyces cerevisiae*. For *C. guilliermondii*, the LD50 (semi-lethal dose) value for X-rays is about 720 Gy (Navasardyan et al., 2017), and for *S. cerevisiae* 300 Gy (Navasardyan et al., 2017) or even 60 Gy (Guo et al., 2018a). The radiation-induced decrease in the percentage of surviving cells leads to the lengthening of the lag phase of the growth of X-ray-irradiated yeast. Further incubation of damaged cells proceeds with DNA repair, and the rate of biomass accumulation gradually increases; by about the end of the first day, the biomass of irradiated yeast can reach the value characteristic of non-irradiated yeast (Navasardyan et al., 2017). The absorbed doses of X-rays used in the present work is sub-semi-lethal dose for *C. albicans*. X-rays gradually inhibit the yeast growth rate within the early stage of the sigmoid curve of their biomass increase but can potentially stimulate repair and proliferation in cells with an intensification of cellular metabolic processes. Recently in a study on the damage-repair response of *S. cerevisiae* following heavy-ion beam irradiation (semi-lethal dose is 80 Gy), it has been established that the peak of the damage-repair response occurred 75 min post-irradiation (Guo et al., 2020). Based on the analyses of multiple biological effects, the phenotypic damages in *S. cerevisiae* cells induced by a semi-lethal dose of X-ray irradiation could be repaired to a great extent within 4 h, and the 0–4 h period is an important time frame to investigate the biological effects induced by semi-lethal doses of X-rays (Guo et al., 2018b). Our results are in agreement with this hypothesis. We observed a significant increase in the nanomotion rate of *C. albicans* 3 to 4 h post irradiation in a sub-semi-lethal dose that can be explained by the transitory increase of the metabolic activity.

The post-irradiation change in metabolic activity can be assessed by monitoring CO_2_ production (Masini et al., 1997). It was revealed that the CO_2_ production by *S. cerevisiae* yeast cells irradiated by soft X-rays decreased in the dose range of 0 to 40 Gy, and for a dose of 2000 Gy it slightly increased. Also, metabolic oscillations (measured by the CO_2_ production of cells) were evidenced in cell suspensions and the oscillation frequencies were changed after the X-ray irradiation (Masini et al., 1997).

In our experiments a significant decrease of the nanomotion of X-ray-irradiated *C. albicans* by 24 h was observed for cell oscillations with frequencies lying in the range of 0.55–1.60 Hz. The radiation-induced decrease of the oscillation amplitude seems to be slightly dependent on the cell cycle phase. The amplitude of the nanomotion rate within the frequency range of 0.55–1.60 Hz for G0-phase cells decreased by 3.37 ± 0.68 times and for G2/S-phase cells by 2.61 ± 0.32 times. The ionizing radiation effects are well-known to be determined by the cell cycle phase. The budding yeast cells are more resistant to radiation inactivation than non-budding yeast (Raju et al., 1972; Langguth and Beam, 1973). As a rule, exponentially growing yeast cells are more resistant to ionizing radiation damage than cells in the stationary phase or G1 (Tippins and Parry, 1982). Both yeast and mammalian cultures *in vitro* are most susceptible to radiation mutations in the early stages of the cell cycle. In the later stages of the cell cycle, yeast cells are most sensitive to the formation of homologous DNA deletion. It was shown that for *S. cerevisiae* within the first 30 h after X-ray irradiation, the magnitude of radiation-induced DNA deletions correlated positively with the fraction of cells in the S/G2 phase (Hafer et al., 2010).

In a second part of this work, we compared the nanoscale oscillatory behavior of *C. albicans* after X-ray irradiation and after antifungal drug treatment. Though FLC exhibits concentration-dependent fungicidal activity, it was shown that no membrane destruction could be observed in a wide range of concentrations (Lee and Lee, 2018). At high concentrations of FLC, both potassium efflux and cell shrinkage have been observed. FLC also causes concentration-dependent apoptotic responses, including phosphatidylserine externalization and DNA fragmentation. It was also involved in glutathione depletion followed by oxidative damage. A high concentration of FLC causes the disruption of mitochondrial homeostasis, including mitochondrial membrane depolarization and accumulation of calcium and reactive oxygen species (ROS) in *C. albicans* (Kobayashi et al., 2002; Wang et al., 2009; Mahl et al., 2015) and in *C. neoformans (* Peng et al., 2018; Dbouk et al., 2019). It was verified that the ROS were involved in FLC-mediated growth inhibition by determining ROS-scavenging proteins and metallothioneins (Dbouk et al., 2019). ROS are generated during basic cellular processes, or due to external stress-inducing conditions, including exposure to X-rays. So, the mechanisms of action of both factors, FLC and X-rays, on *C. albicans* include ROS, and, therefore, the change in the cell nanomotion in both cases may have common features. A temporary increase in the nanomotion rate during the first 4 h for irradiated cells and cells treated with FLC was observed for both treatments. It illustrates a similar cellular response to two very different disturbances. However, there are also obvious differences in the effects of X-ray and FLC on *C. albicans* nanomotion. After exposure to X-ray radiation alone or in combination with FLC, low-frequency nanoscale oscillations of whole cells are significantly suppressed depending on the phase of the cell cycle, absorbed dose, FLC concentration, and post-irradiation period.

There are some limitations of the present work. In this study, we used as a model a *C. albicans* strain that is sensitive to FLC and studied only its planktonic phase. One important characteristic of *C. albicans* is that it can exist in three phases, i.e., planktonic yeast, pseudohyphae, and hyphae phase differing in their cell morphology, function, and growth conditions (Saghrouni et al., 2013; Chen et al., 2020). Yeast cells which are the default cell morphology under most *in vitro* conditions, are round or oval with a unicellular morphology. The choice of the radiation dose used in the work, which is 5–10 times higher than that usually used in radiotherapy in the clinic, was based on the high radioresistance of fungal cells to the action of photons of ionizing electromagnetic radiation such as X-rays. When using ionizing particle beams with high LET, the local doses achieved during a course of radiation therapy (radioimmunotherapy and radionuclide therapy) can be very high and comparable to those critical for the viability of fungal cells (Roosen et al., 2021).

Further development of the study is seen in the expansion of types or strains of fungi, as well as the expansion of the range of doses of X-rays, including those widely used for medical purposes. The potential use of the method in the clinic can be justified by the relatively high speed of evaluation of the sensitivity of *C. albicans* to antifungals and the individual concentration of antifungal drugs in cancer patients undergoing radiation therapy. Since ionizing radiation affects the metabolic activity and functioning of *Candida* cells, a unique combination of ionizing radiation and antifungals can be experimentally determined to achieve the best treatment result with minimal side effects. To implement a clinically applicable method for assessing cellular activity, it is also necessary to conduct a comparative assessment of the parameters of cell nanomotion in parallel with classical methods for assessing cell viability, such as metabolic activity assay (XTT assay) and colony-forming unit counting. The revealed features of the *C. albicans* nanomotion and an understanding of the mechanisms of their change under the action of ionizing radiation and antifungals can help to rapidly determine the sensitivity of *C. albicans* to antifungals and the concentrations of antifungals during repeated courses of radiation therapy for patients with candidiasis.

Moreover, in addition to the medical technology based on the ONMD method discussed in this paper, another technology can potentially be developed, for example, a technology for quality control of irradiation of food products to increase their shelf life and reduce the probability of contamination by microorganisms and toxins that are products of the vital activity of microorganisms, including fungi and mold (Calado et al., 2014).

Long-lasting space flights are well documented to reduce the efficiency of the immune system (Cogoli, 1996; Kelsen et al., 2012) and make astronauts more sensitive to fungal infections (Horneck et al., 2010; Hammond et al., 2013; Senatore et al., 2018; Singh et al., 2018). This technology could rapidly assess sensitivity or resistance to antifungals in microgravity and elevated cosmic radiation environments (e.g., The International Space Station, the Moon, Mars, etc.).

Our work is the first attempt to demonstrate the applicability of the ONMD method for assessing the viability and metabolic activity of fungi cells (*C. albicans*) after the action of ionizing radiation (X-rays) alone and in combination with antifungals. Although the study has a number of limitations, its results allow us to advance in understanding the mechanisms of cell response to ionizing radiation and antifungal agents and their combinations.

## Data availability statement

The original contributions presented in this study are included in the article/Supplementary material, further inquiries can be directed to the corresponding author.

## Author contributions

MS: conceptualization, data analysis, supervision, visualization, and original draft preparation. IC: investigation and data curation. NS: investigation, visualization, data analysis, and software. MV: formal analysis, visualization, and software. DT: conceptualization, data curation, formal analysis, and original draft preparation. RW: conceptualization, supervision, and writing—review and editing. SK: conceptualization, project administration, supervision, draft preparation, and writing—review and editing. All authors contributed to the article and approved the submitted version.

## Footnotes

1
https://www.statskingdom.com/
