# Supplementary material for: Modulation of the nanoscale motion rate of Candida albicans by X-rays
Source: Front Microbiol. 2023 Mar 21;14:1133027. doi: 10.3389/fmicb.2023.1133027 (PMC10070863; doi:10.3389/fmicb.2023.1133027)
Supplement: Supplementary file 1 [file Data_Sheet_1.docx]

Supplementary Material

Modulation of the Nanoscale Motion Rate of *Candida albicans* by X-Rays

Maria N. Starodubtseva^1,2*^, Irina A. Chelnokova^2^, Nastassia M. Shkliarava^2^, María Inés Villalba^3^, Dmitry V. Tapalski^4^, Sandor Kasas^3,5,6,¶^ and Ronnie G. Willaert^6,7,¶^

*** Correspondence:** Maria N. Starodubtseva: **maria.n.starodubtseva@gmail.com**

# Supplementary Table

**Table S1**. The *C. albicans* nanomotion rates (nm/10 ms) depend on the absorbed dose and fluconazole concentration.

|  | **Fluconazole concentration (mg/L)** | | |
| --- | --- | --- | --- |
| **Absorbed dose, Gy** | **0** | **10** | **1000** |
| **0** | 8.38  (5.33,12.48)* | 9.98  (5.99,14.61)*^,#^ | 8.65  (5.44,14.44)** |
| **300** | 7.79  (4.55,11.70)** | 8.85  (5.33,14.39)^#, ###^ | 7.27  (3.87,10.86)*** |
| **600** | 10.26  (6.18,14.28) | 8.37  (5.05,12.98)^#, ####^ | 6.34  (2.86,12.37)^##^ |

Data are presented as Me(LQ;UQ).

Kruskal-Wallis ANOVA by ranks test (p<10^-6^), multiple comparison (Dunn's test) post-hoc analysis: *p<0.004, **p<10^-6^ compared to the parameter at the absorbed dose of 600 Gy and the corresponding concentration of FLC, ***p<10^-6^ compared to the parameter of non-irradiated yeast culture (0 Gy) at the corresponding concentration of FLC, ^#^p<0.004, ^##^p<10^-6^ compared to the parameter at 0 mg/L FLC and the corresponding absorbed dose, ^###^p<0.004, ^####^p<10^-6^ compared to the parameter at 1000 mg/L FLC and the corresponding absorbed dose.

## Supplementary Figures

**Supplementary Figure S1.** The morphology of non-irradiated (A, F, 0 Gy) and irradiated (B-E, 200-800 Gy) *C. albicans* cells. The vials with cells were exposed to X-ray radiation using the X-RAD 320 biological irradiation unit (320 kV, 12.5 mA, 5 Gy/min). After irradiation 20 μl of the cell suspension was dispensed onto a glass slide between two Scotch tapes fixed parallel to each other and perpendicular to the longer side of the slide, then covered by a coverslip. The microscopic images of cells were taken 20 min later the irradiation using MiniMed 502 microscope with a 40× objective.

**
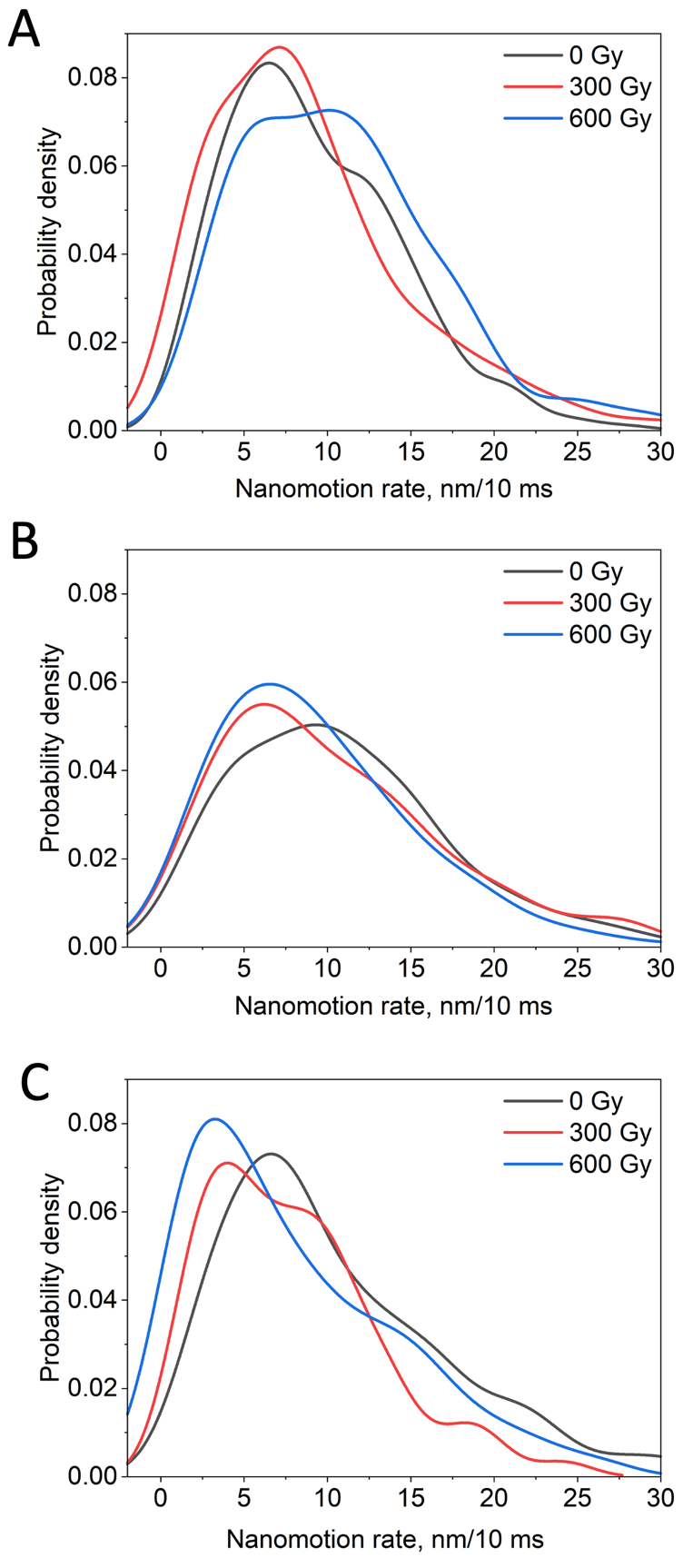
**

**Supplementary Figure S2.** The combined effect of X-rays (0, 300, and 600 Gy) and fluconazole concentration on the distribution of the nanomotion rate of *C. albicans*: (A) 0 mg/L FLC, (B) 10 mg/L FLC, (C) 1000 mg/L FLC.

**Supplementary Video**

**Supplementary Video S1.** Typical video recordings of *C. albicans* cell movement for cell samples (non-budding and budding cells) 24 hours after the start of the experiment. Cell samples: non-irradiated and non-treated cells (control), cells treated with 1000 mg/L fluconazole (FLC), cells irradiated by 600 Gy X-rays (XR), cells treated with 1000 mg/L FLC and irradiated by 600 Gy X-rays (FLC+XR).
